# Vitamin D plasma concentrations in pregnant women and their preterm newborns

**DOI:** 10.1186/s12884-018-2045-1

**Published:** 2018-10-22

**Authors:** Milene Saori Kassai, Fernanda Ramirez Cafeo, Fernando Alves Affonso-Kaufman, Fabíola Isabel Suano-Souza, Roseli Oselka Saccardo Sarni

**Affiliations:** 1 Department of Pediatrics, Faculdade de Medicina do ABC, Avenida Lauro Gomes, 2000. Vila Sacadura Cabral, Santo André, São Paulo 09060-870 Brazil; 2 Department of Pediatrics, Universidade Federal de São Paulo - Escola Paulista de Medicina, Rua Botucatu, 898, Vila Clementino, São Paulo 04023-062 Brazil

**Keywords:** Vitamin D, Newborns, Pregnancy, Umbilical cord

## Abstract

**Background:**

Vitamin D deficiency is a global public health issue. More than half of pregnant women are affected by vitamin D insufficiency/deficiency. Studies suggest an association between low vitamin D concentrations during pregnancy with intrauterine growth restriction and prematurity. This study aimed to describe the concentrations of 25(OH)D (25-hydroxyvitamin D) of mothers who delivered preterm newborns compared to women with full-term pregnancy deliveries, as well as to relate 25(OH)D blood concentrations of mothers with those of their newborns.

**Method:**

This cross-sectional study was conducted with 66 mothers who had given birth to preterm babies and their preterm newborns (PTNB, < 32 weeks), and 92 women who had given birth at the full-term of their pregnancy and their newborns (FTNB). Data were collected on the characteristics of mothers (gestational age, diseases, and habits) and newborns (anthropometry and adequacy for gestational age). Ten milliliters of blood were drawn from the mothers and the umbilical cord of newborns at birth to identify the 25(OH)D, parathyroid hormone, calcium, phosphorus, and alkaline phosphatase concentrations.

**Results:**

Mothers in the PTNB group had significantly lower mean 25(OH)D blood levels (21.7 ± 10.8 ng/mL vs. 26.2 ± 9.8 ng/mL; *p* = 0.011) and were three times more likely to have insufficiency when compared to mothers in the FTNB group (OR = 2.993; 95%CI 1.02–8.74). Newborns in the PTNB group also had lower 25(OH)D concentrations compared to FTNB group (25.9 ± 13.9 ng/dL vs. 31.9 ± 12.3 ng/dL; *p* = 0.009). There was a directly proportional correlation between mother and newborn umbilical cord 25(OH)D concentrations in PTNB (*r* = 0.596; *p* <  0.001) and FTNB (*r* = 0.765; *p* <  0.001).

**Conclusion:**

Mothers who delivered preterm babies and their preterm newborns had lower 25(OH)D concentrations compared to women who had given birth at the full-term of their pregnancy. In both groups, 25(OH)D concentrations of the mothers correlated directly with those of the newborns, and this correlation was higher in the full-term birth group. Nevertheless, the recommended universal vitamin D supplementation in pregnant women to curb the risk of preterm birth is still incipient. More studies are required to clarify the particularities of vitamin D metabolism further and define the adequate 25(OH)D concentrations throughout pregnancy.

## Background

Vitamin D is a prohormone associated with improved bone metabolism and strengthens the immune, respiratory, endocrine and cardiovascular systems [1]. The maintenance of blood concentrations depends predominantly on exposure to sunlight (conversion of cutaneous 7-dehydrocholesterol caused by exposure to ultraviolet B rays) and, to a lesser extent, the intake of vitamin D of either animal-derived (cholecalciferol) or plant-derived (ergocalciferol) food sources. Ethnicity, latitude, season, sunscreen and body mass index also influence vitamin D concentrations [2].

The 25-hydroxyvitamin-D [25(OH)D] and 1,25-dihydroxyvitamin-D [1.25(OH)_2_D] can be measured. However, serum concentrations of 25(OH)D are used both as D2 and D3 to evaluate the nutritional state of patients regarding vitamin D levels. Calcitriol [1.25(OH)_2_D] is the active form, but its half-life is very short and limits patient blood concentration measurement. Vitamin D deficiency can modify bone metabolism markers such as parathyroid hormone, calcium, phosphor and alkaline phosphatase [1, 2].

The 25(OH)D cutoff points currently in use for the general population are > 30 ng/mL, 20 to 30 ng/mL and < 20 ng/dL for sufficiency, insufficiency, and deficiency, respectively, and are based on bone outcomes [3]. Recommended levels are higher than 30 ng/mL for specific groups, such as pregnant women.

Insufficient 25(OH)D levels are a global public health issue, and pregnant women are a significant at-risk group. More than half of pregnant women evidence 25(OH)D concentrations comparable to vitamin D insufficiency [4]. A fetus relies exclusively on the mother’s 25(OH)D concentrations, which affect the placental transfer of calcium and phosphorus, as well as hormonal and immunological balance, all of which are fundamental processes for bone development and fetoplacental integrity [5].

Observational studies suggest correlation between low concentrations of 25(OH)D (25 hydroxyvitamin D) during pregnancy and a higher risk of complications for the mother, such as pre-eclampsia, gestational diabetes, and bacterial vaginosis, as well as complications for the newborn [6, 7], such as intrauterine growth restriction [8], a higher risk of developing allergies [7] and obesity over the long-term [9].

Some international scientific organizations recommend that pregnant women take supplemental doses of vitamin D, ranging from 600 to 1,000 UI daily doses [10–12]. However, the World Health Organization has not universally adopted this practice [13]. The Brazilian Department of Health also does not recommend routine tests for 25(OH)D serum concentrations during prenatal care, nor vitamin D supplements for pregnant women.

Some studies evaluated 25(OH)D concentrations in Brazilian pregnant women [10, 11, 14]. However, no studies have been published on women who have prematurely given birth with a gestational age of fewer than 32 weeks.

This study aims to describe 25(OH)D, parathyroid hormone, calcium, phosphorus and alkaline phosphatase plasma concentrations of women who have delivered preterm (< 32 weeks) compared to women with full-term pregnancy deliveries, as well as to compare the 25(OH)D concentrations in mother with those of their newborn infants in both groups.

## Methods

### Study design

This cross-sectional study was conducted between March 2016 and May 2017 at the Municipal Hospital of São Bernardo do Campo University, São Paulo, Brazil (latitude: 23° 41′ 38” S) with a convenience sample of 66 mothers who had delivered preterm and their newborns (PTNB, < 32 weeks), and 92 mothers full-term pregnancy deliveries and their newborns (FTNB group, 37 to 41 6/7 weeks), who were adequate for gestational age and with a birth weight > 2500 g. The full-term birth group was included during the spring and summer months of the Southern Hemisphere (September 2016 and March 2017).

Mothers with kidney disease, rheumatic disease, diabetes mellitus type 1, acquired immune deficiency syndrome (AIDS), and those using immunosuppressants (such as corticosteroids) were excluded. Newborns with significant malformations, genetic disorders, and neonatal hypoxia were also excluded.

The Research Ethics Committee of the ABC Faculty of Medicine approved the project under opinion N° 1.060.653, dated 13/05/2015. Mothers included in the study agreed to the procedures of the research and signed the informed consent form.

### Data collected

#### Mother’s health and prenatal care

Standardized questionnaires were applied to the mothers, which included questions about their socioeconomic conditions, education, habits (tobacco and alcohol use, medications), pre-existing conditions and obstetric disease (pre-existing pre-pregnancy conditions or those developed during pregnancy), use of vitamin or mineral supplements (iron, folic acid, vitamin D and multivitamins), skin color (white, mixed ethnicity or black), exposure to sunlight, and the regular use of sunscreen.

Their prenatal cards were reviewed for laboratory tests, ultrasounds, date of mothers’ last menstrual period, and the anthropocentric measurements of the fetus during the obstetric monitoring of pregnancy. Pre-pregnancy body mass index (BMI) and weekly weight gain adequacy during pregnancy were based on weight and height measurements (kg/m^2^) [15].

#### Newborn data

Information about the child’s weight, length, and head circumference was retrieved within the first 24 h of life. The gestational age was preferably calculated according to the date of the mothers’ last menstrual period, failing which it was based on the data from the ultrasound taken during the first trimester and, ultimately, according to clinical evaluation of the newborn [16]. The newborns were classified as small, adequate and large for their gestational age using INTERGROWTH-21st standards as a reference [17].

#### Laboratory tests

Blood samples were collected from the mothers on delivery at the obstetrics center. Ten (10) mL of the mothers’ blood were drawn by arm venipuncture and, in the case of the newborns, from the umbilical cord vein. The material was immediately placed in tubes (one dry and one with EDTA) and submitted to the hospital’s clinical analysis laboratory, where they were centrifuged and then transported under refrigeration to the ABC School of Medicine to measure the level of 25(OH)D (electrochemiluminescence, Roche, Mannheim, Germany), intact parathyroid hormone (PTH; electrochemiluminescence, Roche, Mannheim, Germany), calcium, phosphorus, and alkaline phosphatase (colorimetric method). In the case of mothers, 25(OH)D concentrations < 30 ng/mL were considered insufficient, and PTH >  65 pg/mL were deemed to be elevated [3].

All calculations followed the best clinical practice. The mean of 19.9 ng/mL (SD: 0.948 ng/mL and an intra-control CV of 4.8%) was the referred reproducible value to measure the 25(OH)D. For the same parameter, the intermediate precision was 19.9 ng/mL (SD: 1.23 ng/mL and an intra-control CV of 6.2%). For PTH, an average of 54.6 pg/mL was referred for repeatability with SD: 0.657 pg/mL and an intra-control CV of 1.2%. The mean intermediate precision was 54.6 pg/mL with a DP of 1.11 pg/mL and CV of 2.0%.

### Data analysis

A spreadsheet was drafted on Microsoft Excel® with data on identification, general characteristics, data from the questionnaires regarding mothers and newborns, anthropometric data and the results of the laboratory tests. The spreadsheet was revised, consolidated and analyzed using the statistical package SPSS 25.0 (IBM®) to display the results.

The categorical variables were compared using the Chi-squared test in the bivariate analysis. Continuous analyses were tested for their normality through their distribution in histograms and a kurtosis evaluation. Since they were parametric, they were shown according to their means (standard deviation) and compared according to Student’s t-test for independent variables. Pearson’s coefficient was adopted to analyze correlations.

The comparison of the clinical and laboratory variables evidenced that the seasons during which the mothers were pregnant and exposure to sunlight differed between the groups. Thus, a stratified analysis of the frequency of 25(OH)D insufficiencies during spring and summer was conducted with multivariate analysis, using a logistic regression according to the Enter mode method to assess the adjusted effect of prematurity on the odds ratios of 25(OH)D insufficiency.

Pearson’s correlation assessed the correlation between mothers’ and newborns’ 25(OH)D concentrations in both groups assessed by Pearson’s correlation. The significance level adopted was 5%. The sample employed in the study proved sufficient to detect a difference of 5 ng/mL 25(OH)D between the groups [18] by adopting bidirectional α = 0.05 and β = 0.20.

## Results

Table 1 shows the general characteristics of the mothers and newborns in both the preterm and full-term birth groups. There was no difference between the groups regarding mother’s age, ethnicity, number of pregnancies, sunscreen use, alcohol use, tobacco use, pre-pregnancy body mass index, or use of vitamin D or iron supplements. Mothers in the PTNB group had a lower frequency of folic acid supplementation [Yes: 46 (70.8%) vs 92 (87.6%); *p* = 0.038], prenatal care [< 6 visits: 54 (81.8%) vs 20 (21.7%); *p* <  0.001) and regular exposure to sunlight [Yes: 5 (7.6%) vs 54 (58.7%); *p* <  0.001].

**Table 1:** Description of the general characteristics of the mothers, preterm newborns (PTNB) and full-term newborns (FTNB)

Variables		PTNB Group	FTNB Group	*p-value*
		*N* = 66	*N* = 92	
Characteristics of the mothers				
Age (*n* = 158)	Years	26.0 ± 7.3	26.03 ± 6.8	0.987^a^
Ethnicity (*n* = 158)	White	18 (27.3%)	35 (38.0%)	0.151^b^
	Black	6 (9.1%)	3 (3.3%)	
	Mixed ethnicity	42 (63.6%)	54 (58.7%)	
Schooling (*n* = 150)	< 4 years	5 (8.5%)	2 (2.2%)	0.194^b^
	4 to 8 years	50 (84.7%)	81 (89.0%)	
	> 8 years	4 (6.8%)	8 (8.8%)	
Number of pregnancies (*n* = 158)	Number	2.2 ± 1.5	2.3 ± 1.5	0.761^a^
Alcohol use (*n* = 158)	Yes	5 (7.6%)	4 (4.3%)	0.492^b^
Tobacco use (*n* = 158)	Yes	13 (19.7%)	10 (10.9%)	0.169^b^
Vitamin D Suplementation (*n* = 158)	Yes	6 (9.1%)	5 (5.4%)	0.528^b^
Folic Acid (*n* = 158)	Yes	46 (70.8%)	92 (87.6%)	0.038^b^
Iron (*n* = 158)	Yes	50 (75.8%)	76 (82.6%)	0.320^b^
Frequent use of sunscreen (*n* = 158)	Yes	9 (13.6%)	23 (25.0%)	0.108^b^
Regular exposure to sunlight (*n* = 158)	Yes	5 (7.6%)	54 (58.7%)	< 0.001^b^
Pregnancy-induced illness (*n* = 158)	Hypertension	27 (40.9%)	14 (15.2%)	< 0.001^b^
	Gestational diabetes	3 (4.5%)	2 (2.2%)	0.650^b^
	Urinary tract infection	14 (21.2%)	32 (34.8%)	0.077^b^
Prenatal care (*n* = 158)	Yes (> 6 check-ups)	12 (18.2%)	71 (78.3%)	< 0.001^b^
	Yes (< 6 check-ups)	42 (63.6%)	16 (17.4%)	
	No (no check-ups)	12 (18.2%)	4 (4.3%)	
Pre-pregnancy BMI (*n* = 116)	> 30 kg/m^2^	6 (19.4%)	19 (22.4%)	0.905^b^
	25 to 29.9 kg/m^2^	8 (25.8%)	22 (25.9%)	
	18.5 to 24.9 kg/m^2^	16 (51.6%)	39 (45.9%)	
	< 18.5 kg/m^2^	1 (3.2%)	5 (5.9%)	
Pregnancy weight gain	High	18 (58.1%)	55 (65.5%)	0.720^b^
	Adequate	7 (22.4%)	14 (16.7%)	
	Low	6 (19.4%)	15 (17.9%)	
Season (*n* = 158)	Spring	14 (21.2%)	48 (52.2%)	< 0.001^b^
	Summer	24 (36.4%)	44 (47.8%)	
	Fall	16 (24.2%)	0 (0.0%)	
	Winter	12 (18.2%)	0 (0.0%)	
Newborn Characteristics				
Gender (*n* = 158)	Male	35 (53.0%)	50 (54.3%)	0.727^b^
Type of birth (*n* = 158)	Vaginal	17 (25.8%)	48 (52.2%)	0.001^b^
Gestational Age (*n* = 158)	Weeks	29.8 ± 2.4	39.3 ± 1.2	< 0.001^a^
Birth weight (*n* = 158)	Grams	1250 ± 354.6	3317 ± 449.4	< 0.001^a^
Size for gestational age (*n* = 158)	Small for GA	14 (21.2%)	0.0 (0.0%)	< 0.001^b^
	Adequate for GA	47 (71.2%)	83 (90.2%)	
	Large for GA	5 (7.6%)	9 (9.8%)	

^a^Average and standard deviation. Level of significance of Student’s t-test

^b^Number (percentage). Level of significance of the chi-squared test

The main complications associated with premature births were pregnancy-related hypertensive disorders (27) (40.9%; *p* <  0.001). Among women with pregnancy-related hypertensive disorders, 21 (77.8%) were taking methyldopa.

The mean of gestational age and birth weight in newborns of the PTNB group were 29.8 ± 2.4 weeks (*p* <  0.001) and 1250 ± 354.6 g (*p* <  0.001), respectively. In this group, 14 (21.2%) were small for their gestational age (Table 1). All these variables show a statistically significant difference concerning the FTNB group (Table 1).

The mothers in the PTNB group had lower mean values (21.7 ± 10.8 ng/mL vs. 26.2 ± 9.8 ng/mL, *p* = 0.011) and higher percentage of 25(OH)D insufficiency and deficiency when compared with the FTNB group (*p* = 0.018) (Table 2). Logistic regression showed that mothers who had delivered preterm babies were three times more likely to have insufficient 25(OH)D (OR = 2.993; 95%CI 1.02–8.74) (Table 3).

**Table 2:** Description of general characteristics of preterm newborns (PTNB) and full-term newborns (FTNB) included in this study

Variables		PTNB Group (*n* = 66)	FTNB Group (*n* = 92)	*p*-value
Mother				
25(OH)D (*n* = 144)	ng/mL	21.7 ± 10.8	26.2 ± 9.8	0.011^a^
Deficiency	n (%)	27 (43.5%)	23 (28.0%)	0.018^b^
Insufficiency	n (%)	25 (40.3%)	29 (35.4%)	
Sufficiency	n (%)	10 (16.1%)	30 (36.6%)	
Parathyroid hormone (*n* = 121)	pg/mL	50.1 ± 40.7	51.1 ± 37.6	0.890^a^
Calcium (*n* = 145)	mg/dL	7.8 ± 1.2	8.7 ± 1.0	< 0.001^a^
Phosphorus (*n* = 144)	mg/dL	3.4 ± 1.5	3.3 ± 0.9	0.718^a^
Alkaline phosphatase (*n* = 145)	U/L	178.0 ± 81.6	269.1 ± 77.4	< 0.001^a^
Umbilical cord				
25(OH)D (*n* = 140)	ng/mL	25.9 ± 13.9	31.9 ± 12.3	0.009^a^
Deficiency	n (%)	25 (41.7%)	17 (21.3%)	0.033^b^
Insufficiency	n (%)	13 (21.7%)	24 (30.0%)	
Sufficiency	n (%)	22 (36.7%)	39 (48.4%)	
Calcium (*n* = 139)	mg/dL	9.3 ± 1.1	10.2 ± 1.0	< 0.001^a^
Phosphorus (*n* = 138)	mg/dL	5.9 ± 2.1	5.0 ± 1.2	0.002^a^
Alkaline phosphatase (*n* = 140)	U/L	363.3 ± 185.1	324.1 ± 344.4	0.431^a^

^a^Level of significance of the Student’s t-test. ^b^Level of significance of Chi-square test

**Table 3:** Odds ratio of 25(OH)D insufficiency in the mothers included in this study

Variable	Beta	Confidence Interval 95%	*p*-value
Season (Spring/Summer)	0.831	0.21 to 3.30	0.793
Exposure to sunlight (no)	1.158	0.48 to 2.74	0.739
Prematurity	2.993	1.02 to 8.74	0.045

No correlation was observed between 25(OH)D insufficiency and pregnancy complications (PIH, DM, and m UTI) nor habits during pregnancy (tobacco use, alcohol use, and iron, folic acid, and vitamin D supplementation) among women in the PTNB group (data not shown).

The 25(OH)D levels of newborns were also lower in the PTNB group compared to the FTNB group (25.9 ± 13.9 ng/dL vs. 31.9 ± 12.3 ng/dL; *p* = 0.009) (Table 2). Vitamin D insufficiency and deficiency was higher in the PTNB group compared to the FTNB group (*p* = 0.033) (Table 2).

**Fig. 1:**
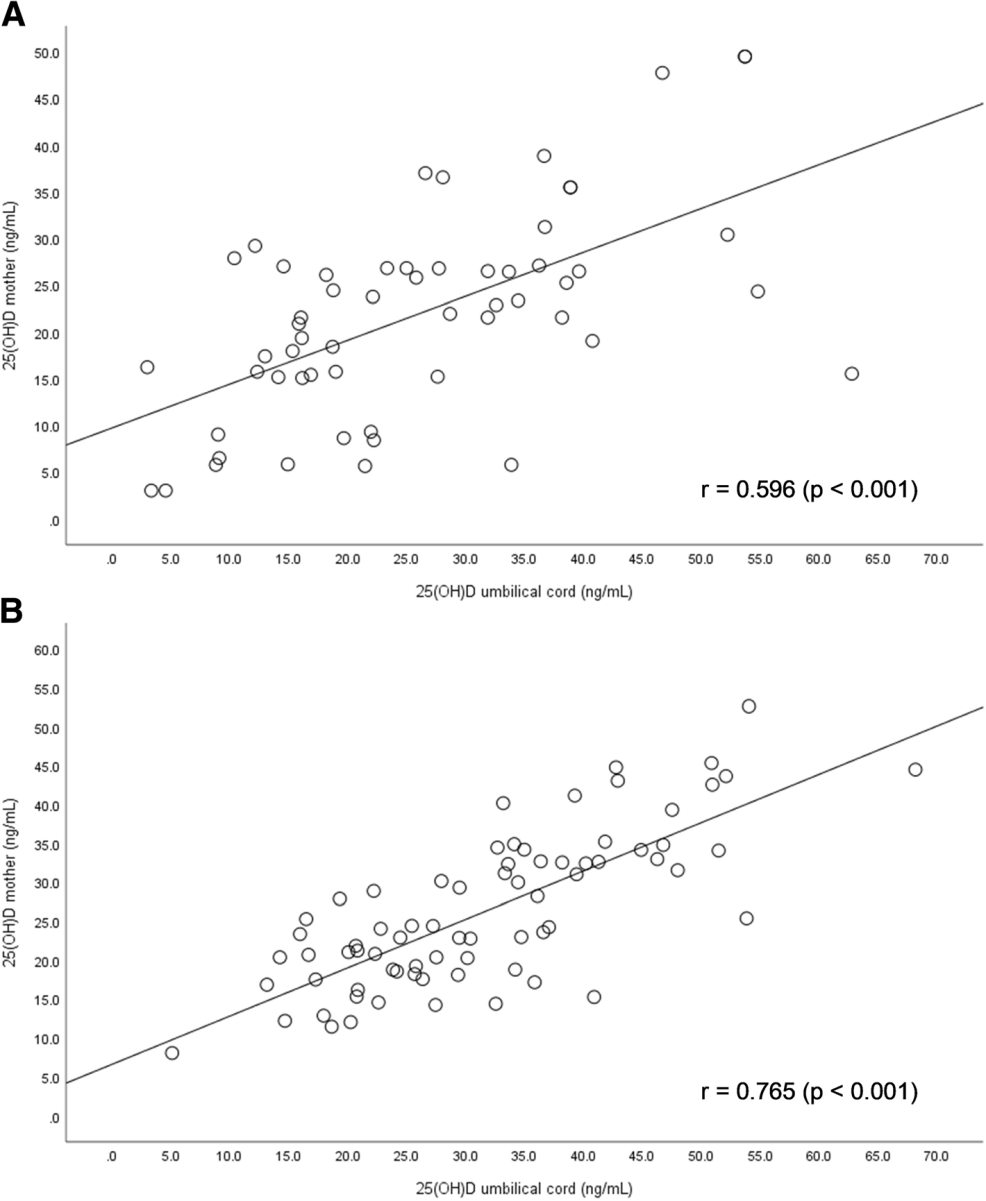
Correlation of 25(OH)D concentrations in the mothers and umbilical cords in the PTNB group (**a**) and FTNB group (**b**). Level of significance of Pearson’s correlation test

There was a directly proportional correlation between 25(OH)D concentrations of mother and newborn umbilical cord in the PTNB group (*r* = 0.596; *p* <  0.001) and the FTNB group (*r* = 0.765; *p* <  0.001) (Fig. 1). An inverse correlation was found between PTH and 25(OH)D concentrations in all mothers (*r* = − 0.230; *p* = 0.012). However, no statistically significant difference was observed between the groups concerning PTH (23.2% vs. 12.3%; *p* = 0.090).

With regard to the other bone metabolism-related markers, we found that the PTNB group had lower levels of calcium (7.8 ± 1.2 mg/dL vs. 8.7 ± 1.0 mg/dL; *p* <  0.001) and alkaline phosphatase (178.0 ± 81.6 U/L vs. 269.1 ± 77.4 U/L; *p* <  0.001) when compared to the FTNB group (Table 2).

## Discussion

This study found that mothers who had given birth prematurely had a lower concentration of 25(OH)D compared to those who had given birth the end of their full term. The 25(OH)D levels of the newborns, in both groups, correlated with those of the mothers. However, this correlation was more significant in the FTNB group.

Vitamin D sufficiency (> 30 ng/mL) was found in 20.8% of mothers and 42.3% of newborns. Some studies in Brazil describe the 25(OH)D concentrations during pregnancy [10, 11, 14]. The prevalence of insufficiency/deficiency evidenced by them (range: 44.1% to 58.9%) was lower than this study, taking to account term birth (63.4%) and preterm birth (83.9%). Lower exposure to sunlight, no intake of supplements, low socioeconomic level, and African descent are considered at risk for insufficiency [4]. There are currently no data available for Brazil regarding preterm newborns and their mothers.

The cross-sectional study model does not allow the attribution of a cause and effect relationship. Therefore, we cannot infer whether a vitamin D deficiency was the cause of preterm birth. However, a meta-analysis that included only longitudinal studies confirmed that mothers with 25(OH)D concentrations below 30 ng/mL were 83% more likely to give birth prematurely [19].

The transport and metabolism of vitamin D of mothers and newborns have several particularities that should be considered when interpreting the results of this study. First, vitamin D is transported in the blood by the vitamin D-binding protein (DBP) (90%) and albumin (10%), and only 0.03% of it is unbound. The liver increases its DBP production, and lower levels of circulating albumin [20] are observed during pregnancy. The method adopted in this study to analyze 25(OH)D only allows for the evaluation of the DBP-bound percentage.

Secondly, the production of epimers related to the metabolism of vitamin D [ex: 3-epi25(OH)D3 and 3-epi-1.25α(OH)_2_D3] rise during pregnancy, as well as in infants during the first 3 months of life [21]. This elevation is more significant in preterm newborns [21]. Until recently, it was not possible to measure the level of epimers in the blood or understand their function. However, recent studies have shown their affinity with 25(OH)D receptors, as well as 1,25(OH)D receptors, and also inhibit the production of PTH. Therefore, they play a role in the metabolism of vitamin D situations, such as prematurity [22].

Studies have shown the benefits of taking vitamin D supplements to reduce the probability of preterm birth [6]. In 2016, Wagner et al. observed a 60% decline in premature births in U.S. mothers with 25(OH)D concentrations above 40 ng/dL. The authors concluded that a prescribed supplementation of at least 4000 UI/day was required [23] to achieve these levels.

PTH concentrations are influenced by dietary factors (calcium intake), 1,25(OH)D levels and peptide levels related to PTH (PHTrP) produced in the parathyroid of the fetus and the placental tissue, and that step up vitamin D synthesis. While no difference in the mean PTH levels was observed between the groups, inverse correlation was found between PTH and 25(OH)D concentrations in all mothers. From a physiological perspective, the opposite was expected, as PTH levels tend to increase in the third trimester of pregnancy [24, 25]. A study that assessed the development of PTH concentrations in pregnant adolescents found an increase of 16.3 pg/mL between the 26th week of pregnancy and the moment of birth [26].

A difference between the levels of calcium, phosphorus and alkaline phosphatase in the blood was found between mothers who delivered preterm babies and those with full-term pregnancy deliveries. However, none of these markers correlated significantly with the 25(OH)D concentrations found in newborns and their mothers, suggesting that the factors involved in this alteration may be more related to maternal hemodilution than to vitamin D [27].

The 25(OH)D concentrations found in the mothers were lower than in their newborns and correlated with those of the newborns in both groups. In the meta-analysis published by Saraf et al., 2016 [4], forty studies confirmed a correlation between 25(OH)D concentrations in mothers and the umbilical cords of newborns, and the correlation coefficient ranged from 0.42 to 0.96. Only three studies found higher levels in the umbilical cords than in the mothers. According to the findings of this study, the closer a mother is to the end of her full-term pregnancy, the stronger the correlations of vitamin D levels between mother and newborn will be. All the three studies that found higher levels in newborns showed a higher prevalence of vitamin D insufficiency/deficiency. These findings suggest that gestational age and vitamin D sufficiency interact with the flow of vitamin D from mother to newborn.

Given the particularities of vitamin D metabolism during pregnancy, the lack of well-established cut-off points, and the limited number of studies evaluating the short and long-term safety of supplementation for mothers and their children, it is not currently advisable to universally recommend vitamin D supplementation to pregnant women. The identification of at-risk expecting mothers and the measurement of other vitamin D metabolism markers can provide clues regarding which pregnant women indeed evidence low levels and should take supplements.

Recently, a randomized, double-blind, placebo-controlled trial in Bangladesh assessed the effects of weekly prenatal vitamin D supplementation during pregnancy and postpartum. The authors did not find improvement in fetal or infant growth until 1 year of age [28].

To date, this is the first study conducted in Brazil that has evaluated 25(OH)D concentrations in a group of mothers who gave birth before the 32nd week of pregnancy and their newborns.

The lack of an evaluation of dietary factors, especially mothers’ calcium intake, the difficulty of collecting part of the prenatal care data and accurately establishing the cause of preterm delivery can all be considered limitations of this study.

## Conclusions

In conclusion, mothers who had delivered preterm babies (less than 32 weeks) and their premature newborns had lower 25(OH)D concentrations compared to women with full-term pregnancy deliveries. In both groups, 25(OH)D concentrations of the mothers correlated directly with those of the newborns, and this correlation was higher in the full-term birth group. Nevertheless, the recommended universal vitamin D supplementation in pregnant women to curb the risk of preterm birth is still incipient. More studies are required to clarify the particularities of vitamin D metabolism further and define the adequate 25(OH)D concentrations throughout pregnancy.

## Abbreviations

25(OH)D: Vitamin D
FTNB: Full-term newborn
PTH: Intact parathyroid hormone
PTNB: Preterm newborn
